# A Multiscale Modelling Approach for Estimating the Effect of Defects in Unidirectional Carbon Fiber Reinforced Polymer Composites

**DOI:** 10.3390/ma12121885

**Published:** 2019-06-12

**Authors:** Kim-Niklas Antin, Anssi Laukkanen, Tom Andersson, Danny Smyl, Pedro Vilaça

**Affiliations:** 1 Department of Mechanical Engineering, Aalto University, Puumiehenkuja 3, 02150 Espoo, Finland; kim-niklas.antin@aalto.fi; 2 VTT Technical Research Centre of Finland, Kivimiehentie 3, 02044 VTT, Finland; anssi.laukkanen@vtt.fi (A.L.); tom.andersson@vtt.fi (T.A.); 3 Department of Civil and Structural Engineering, University of Sheffield, Mappin Street, Sheffield S13JD, UK; d.smyl@sheffield.ac.uk

**Keywords:** modelling, carbon fiber composite, experimental mechanics, multiscale, defect

## Abstract

A multiscale modelling approach was developed in order to estimate the effect of defects on the strength of unidirectional carbon fiber composites. The work encompasses a micromechanics approach, where the known reinforcement and matrix properties are experimentally verified and a 3D finite element model is meshed directly from micrographs. Boundary conditions for loading the micromechanical model are derived from macroscale finite element simulations of the component in question. Using a microscale model based on the actual microstructure, material parameters and load case allows realistic estimation of the effect of a defect. The modelling approach was tested with a unidirectional carbon fiber composite beam, from which the micromechanical model was created and experimentally validated. The effect of porosity was simulated using a resin-rich area in the microstructure and the results were compared to experimental work on samples containing pores.

## 1. Introduction

Fiber-reinforced polymers are important materials for structural applications in many fields. For example, there are several notable applications of unidirectional (UD) carbon fiber reinforced polymers (CFRP) in civil engineering [1,2,3,4]. Various mathematical regimes, including analytical [5,6], semi-analytical [7], and numerical models [8] have been formulated to estimate the strength of these materials [9]. Homogenised macroscale models [10] do not correctly capture the failure mechanisms of a composite material and therefore micromechanical models have been developed as an alternative [11]. Micromechanical models can be divided into phenomenological models, such as shear lag and fiber bundle models [12], and numerical models using the finite element method (FEM) [9]. Although both types of micromechanical models take into account the interactions between fiber and matrix, only numerical models have the potential to fully capture the complex nature of damage evolution in composites [13]. They have the capability of accurately describing how failure starts and how defects that are small compared to the microstructural features affect the performance of a composite. Numerical micromodels can also be used to solve ply properties without experimental work when designing laminates. Several analytical models have been developed for solving the homogenised properties based on constituent properties [14]. However, the analytical models do not always perform reliably, especially when fiber volume fractions are higher than 0.6 [15]. The analytical solutions are, however, simple to use. These homogenised macroscopic material properties are needed as an input for computation of mechanical finite element models. Micromechanical modelling is useful during the service life of a component as well. It could provide a means to simulate the response of a structure as a function of different sizes and types of damage occurring during service, and hence be integrated in relevant condition monitoring systems. It also allows for continued assessment and provides better information as to the required actions when damage is found and characterized via non-destructive evaluation [16]. In other words, it furthers the ability to determine the residual strength and evaluate whether or not a component can continue in service. However, micromechanical models require the correct input, namely fiber and matrix properties (constituent properties) and microstructure, in order to deliver reliable results.

Obtaining constituent properties and validating the homogenised output is not straightforward for anisotropic composite materials. However, transversely isotropic materials like carbon fiber and unidirectional fiber-reinforced composites have only five independent material constants [17]. The longitudinal Young’s modulus of the fiber is straightforward to calculate from instrumented tensile tests [18,19]. Furthermore, the rule of mixtures is well established for axial properties of UD composites and allows inverse micromechanics to be used with confidence. Direct measurements of transverse Young’s modulus E_2f_ have been conducted using nanoindentation [14] and the results were found to be slightly higher than those obtained using various analytical inverse micromechanics solutions [20]. Resonance frequencies have also been used to measure transverse Young’s modulus of carbon fibers [21]. Obtaining experimentally, the remaining three elastic constants of a single filament is challenging. Many authors resort to measuring composite properties and using inverse micromechanics [22]. Some modelling papers simply assume certain elastic properties [22,23,24] while others cite sources that are difficult to obtain [25].

The modelled microstructure should describe information such as fiber volume fraction, packing, size and shape as accurately as possible. Periodic square or hexagonal fiber packing is an idealized case whereas random packing generators create a more realistic distribution of fibers [26], but high fiber volume fractions are challenging for the generators [27]. There is an effect on transverse elastic constants between periodic and random packing [15]. In strength models, fibers very close to each other can lead to a significant increase in stress concentration factors [26], but the stress recovery distance is smaller due to a locally stiffer matrix. Hence, packing type does not make much of a difference for uniaxial loading of UD CFRPs in the fiber direction [28]. However, matrix plasticity and debonding may change results [29] and off-axis loading requires random packing for accurate results [30]. 

This paper describes the creation of a multiscale microstructure-based model and its experimental validation. Ultimately, the goal of this work is to create a macroscale strength model that considers microstructural defects without the need for experimental model updating or calibration. The structure of the paper is as follows: First, the general structure of the multiscale approach and the pre-requisites for a micromechanical model are described. Second, the numerical methods for calculating composite properties using the information established in the previous step are presented. Third, the composite properties are used to obtain relevant boundary conditions (BCs) for loading the original micromodel and simulating the effect of a defect on strength. The modelling results are compared to measurements of a pultruded UD CFRP beam at each step.

## 2. Materials and Methods 

**Figure 1:**
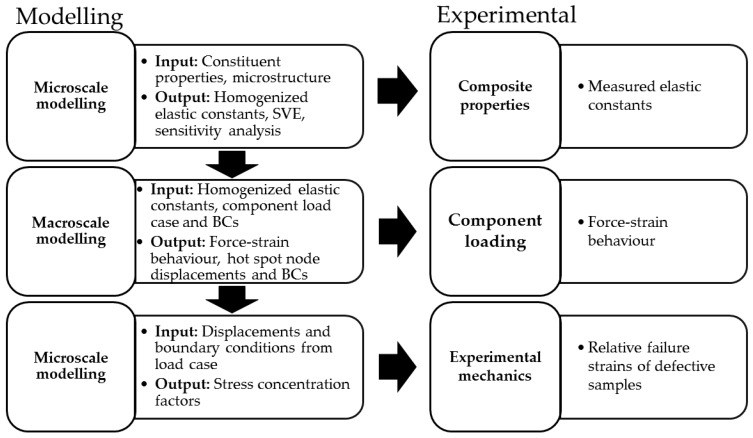
Flow chart describing the modelling process with concurrent experimental work.

The workflow (Figure 1) follows a typical multiscale approach [31] where microscopic behaviour is described using a representative volume element (RVE) and global response is simulated using homogenisation of the RVE (first step). Statistical representation of the microstructure is evaluated by varying the RVE size until stabilization of homogenised properties occurs, i.e., a statistical volume element (SVE) is obtained. The sensitivity of the homogenised properties to changes in constituent properties, i.e., fiber and matrix properties, are analysed with the aim of assessing the importance of individual input parameters, since not all of them are necessarily well known. Homogenised properties can be measured experimentally [32], but the goal here is that no experimental calibration [33] or inverse micromechanics is used. There are two reasons for this: First, experimental work can be expensive and time-consuming if it is needed every time an input parameter changes. Second, using inverse micromechanics or model calibration makes experimental validation redundant. The second step in the workflow is to insert the homogenised properties into a macroscopic model, which takes the component geometry, boundary conditions and load cases into consideration. The response of the macroscopic model can be validated experimentally by loading the component and comparing measured strain values with simulated strain. The macroscopic model is used to identify critical areas in the structure. The third step in the workflow involves using the critical locations for defining displacements and boundary conditions for the RVE so that they are relevant with the practical application in mind. The effect of known defects found using advanced non-destructive testing [16] or postulated defects can now be evaluated in microscale with loading conditions relevant to real-life applications. The simulated failure strength of a defective component can thus be calculated and compared to experimentally obtained failure loads. Ideally, this process allows the estimation of residual strength of a defective component based on in-service inspection results.

The macroscopic model and case study presented in this paper is three-point bending of a pultruded UD CFRP beam. Constituent properties and micrographs are used to create the RVE and to obtain homogenised composite properties. The critical location is identified from the macroscopic simulation results and the node displacements at that location are used as a load case for the RVE to evaluate the effect of porosity on the strength of the beam. Experimental validation is done for each step.

### 2.1. Constituent Properties

**Figure 2:**
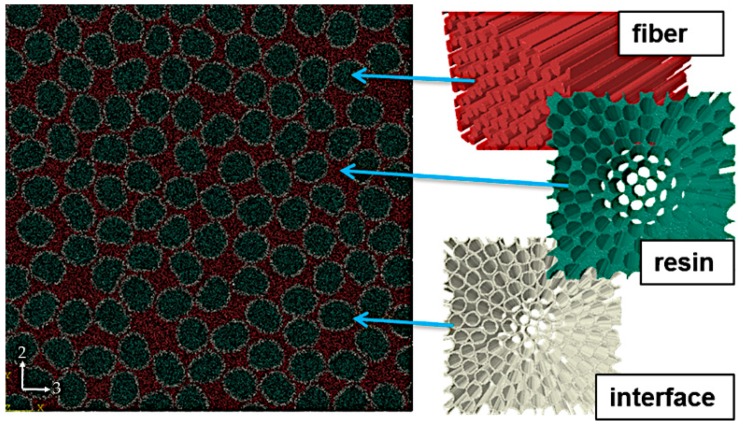
Representation of the three-phase microstructure: algorithmically detached fibers, interface (interphase) added to the fiber perimeters and resin filling the rest.

The CFRP rods were manufactured at an industrial production plant using a heated pultrusion die. The composite constituents are standard modulus (high strength) polyacrylonitrile-based (PAN) carbon fiber reinforcement and epoxy resin matrix. Constituent properties given by the CFRP manufacturer are used in this study, which is a typical source in modelling papers [34,35]. This paper uses two Young’s moduli E_1_ and E_2_, one shear modulus G_12_ and two Poisson’s ratios ν_12_ and ν_23_. These are the most feasible material constants to obtain experimentally. Here, fiber direction is denoted as “1” and the transverse plane as “2-3” (Figure 2). 

Experimental verification of the given constituent properties were conducted where possible using instrumented nanoindentation. Indentation was performed using a CSM Instruments MCT tester (Needham, MA, USA) on longitudinal and transverse cross-sections of the UD CFRP material with the intention of verifying E_1f_, E_2f_ and E_m_, where the subscripts “f” and “m” stand for fiber and matrix, respectively. Specimen cross-sections were wet sanded to FEPA P4000 grit and ten measurements were made in a line with 10 µm intervals. An indentation depth of 0.1 µm was selected because the indentation modulus stabilizes at relatively high values [14,36,37]. On the other hand, deeper indentation was avoided in order to keep the area function of the sphero-conical tip continuous and to avoid fracture. It was apparent from the results which indentations had hit the fiber and which were on the matrix. For the transverse sample, the direction of the measurement line was perpendicular to fiber direction meaning that no two measurements are from the same filament. The indentation parameters are: Indenter = SB-B28 sphero-conical; Tip radius = 2 µm; Cone full angle = 90°; Indentation depth = 0.1 µm; (un)Loading rate = 0.8 mN/min; Dwell time = 30 s; Data acquisition rate = 10 Hz. 

The initial unloading slope was determined from the force-displacement data. The typically used power-law fit proposed by Oliver & Pharr [38] did not produce high-correlation fits and therefore a quadratic polynomial was used. Any permanent displacement (h_f_) was subtracted from the data and intersection with the origin was imposed. The derivative of the polynomial fit at maximum displacement was used to obtain the initial unloading slope or contact stiffness, S [39]. The contact stiffness was used to calculate the contact depth (h_c_) using parameter ε = 0.75 as proposed in [38]. The contact depth was used to calculate the projected contact area and thus the indentation modulus M as defined by Vlassak [40]. The indentation modulus of the isotropic matrix is straightforward to calculate using the Oliver & Pharr method [38] when the indenter properties are known. For an anisotropic material, where the contact area is elliptical, another solution is used [41,42]. The principle there is to solve all five stiffness constants using a five-equation system by inserting three previously known stiffness constants and two perpendicular indentation results. A one-at-a-time sensitivity analysis showed that none of the inserted engineering constants alone affects the results to a significant extent. The sensitivity analysis was conducted by doubling or halving each engineering constant one at a time. The resulting values for E_2f_ were maintained within 10% of the reference case. The indentation modulus had the largest effect, which was close to a linear dependency.

### 2.2. Microstructure

Since the ultimate goal here is a strength model, real microstructure of a pultruded CFRP beam is used to generate the model morphology. High-resolution X-ray microtomography has been conducted on the pultruded CFRP material [16]. However, distinguishing between fiber and matrix from microtomography voxel data was found to be unreliable and therefore a 2.5D approach was selected. Completely straight fibers are assumed although the tomography data [16] and transverse cross-sections [3] indicate some fiber waviness. Imaging of 2D cross-sections was made using a Hitachi SU1510 variable pressure scanning electron microscope (VP-SEM) (Tokyo, Japan) and backscatter electron (BSE) detection. The incident electrons were accelerated with a potential of 25 kV in order to get a higher yield of back-scattered electrons compared to lower acceleration voltages. Fiber volume fraction is analysed from that image as well using a binary colour map and manually adjusted threshold criteria. Defects were introduced to the pultruded material by adding water to the resin bath at the pultrusion line. The resulting pore content cannot be controlled due to the differing density between water and resin, heterogeneous dispersion and the continuous nature of the pultrusion process. Consequently, optical microscopy had to be used to characterize the resulting pore content and typical pore size. A Nikon Epiphot 200 microscope (Tokyo, Japan) was used and images were recorded with a Nikon DigitalSight DS-U1 camera (1600 × 1200 px). The 2.5D approach for generating a 3D mesh does not allow using direct image-based meshing for the microstructure containing pores. Instead, a resin-rich area of the microstructure is used to represent the effect of porosity.

### 2.3. Microscale Modelling

An image-based approach is used to obtain a representative microstructure for further micromechanical analysis. SEM images were segmented initially to a two-phase depiction of the material, the fiber and resin phases, respectively. In order to improve the realism of modelling and eliminate possible artefacts from SEM specimen preparation, individual fibers were detached algorithmically. These two-dimensional segmented images were extruded to yield a cubic representative volume element (RVE) of the composite. As image-based meshing was utilized; no geometric representative of the microstructure was generated at any point, but rather, the segmented data is meshed directly. In addition to the fiber and resin phases, an interface region is included (interphase) (Figure 2). The strategy chosen in the current work is to include the interface firstly to obtain separation of individual fibers and secondly to yield a better description of the composite microstructure and interaction between the fibers and the resin [43,44]. The approach falls within effective interface approaches, i.e., the interface is a third phase, which effectively captures the interface region behaviour between fibers and resin by employing its own mechanical material properties. Further details of the modelling toolset utilized in creating the interphase are presented in [31].

Numerical homogenisation was employed in determining the engineering material properties of the composite based on micromechanical modelling results. The RVEs were loaded under kinetic-uniform boundary conditions (KUBC) and subjected to differing imposed strain states to compute the volume averaged metrics for solving the composite material properties. In addition, the computational volume from which the data was extracted was considered a variable in order to ascertain that the RVE size is representative of composite behavior. This was carried out by sampling increasing material volumes beginning from the center of the microstructure towards its external boundaries and assessing the changes in material property predictions.

The homogenised composite properties obtained with microscale numerical modelling were compared to analytical micromechanical equations. Results using the equations by Chamis [20] are included as a comparison, since those equations require only the fiber volume fraction in addition to the constituent properties. Other models often require some empirical parameters for the material in question [17], which is effectively model calibration.

All of the homogenised composite properties were verified experimentally. The parameters E_1_ and ν_12_ were measured in uniaxial tensile and compression loading using an MTS 810 servohydraulic machine (Eden Prairie, MN, USA) with a 100 kN load cell following the procedures in ISO 527-5 [45], with Kyowa strain gauges bonded parallel and perpendicular to fiber direction. The parameter ν_12_ was solved by linear regression of the ε_t_-ε_L_ strain data instead of measuring transverse thickness of the sample as is suggested in the standard. Transverse compression was used to obtain E_2_ and ν_23_. Strain was calculated using DaVis 8.1 software by LaVision Gmbh from micro-DIC (Digital Image Correlation) measurements (Goettingen, Germany) done with LaVision Imager ProX 2M camera (1600 × 1200 px). The fibers were used as contrast pattern for image correlation. Furthermore, quasi-static elasticity imaging was used to solve E_1_, E_2_, ν_12_ and G_12_. All of the previous macroscale experiments are explained in more detail in [46].

### 2.4. Macroscale Modelling

**Figure 3:**
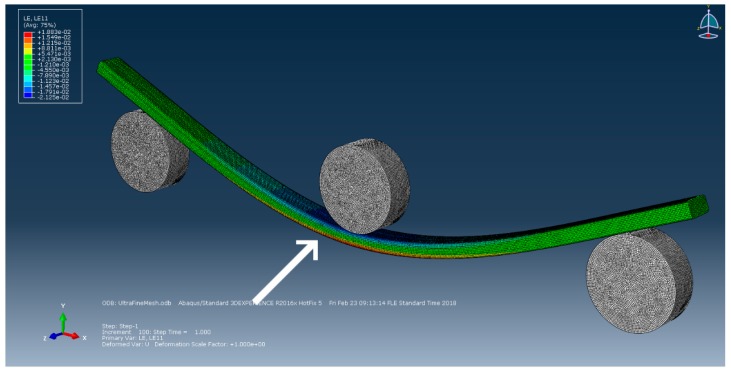
Location of strain hot spot in simulated three-point bending.

The finite element method was used for simulating the macroscale behaviour of the UD CFRP beam. The simulations allow experimental verification of the behaviour of a real component using the material constants obtained in the previous step. In addition, they give boundary conditions (BCs) and node displacements for the micromodel that are relevant to a real loading situation. The three-point bending setup used for experimental work was modelled and meshed in Abaqus CAE using quadratic tetrahedral (C3D10) elements (Figure 3). The rollers were defined as rigid shell bodies with Hertzian contact and a 0.15 friction coefficient [47]. Orthotropic material properties from the homogenised micromechanical model were used for material properties of the specimen. A load of 658 N was applied to the central roller, which corresponds to the forces seen in earlier experimental work [46]. In addition, cases with isotropic assumptions and a sensitivity analysis to individual material parameters was made. The results were compared to flexural tests according to ISO 14125 [48] and apparent interlaminar shear strength (ILSS) tests according to ISO 14130 [49]. Both bending experiments used the same MTS 810 testing system as the uniaxial tests.

## 3. Results

The results are presented following the simulation workflow. First, constituent properties given by the manufacturer are compared to measured values. The micrographs used for meshing are also presented. Second, the representative microstructure is created and homogenised to obtain composite properties. The results are compared to those obtained by analytical and experimental methods. Third, macroscale simulations are used to create relevant loads for the micromechanical model. Last, CFRP components are tested for failure and compared to the simulated stresses and strains of corresponding microstructures.

### 3.1. Constituent Properties

The micromodel inputs are the constituent properties and microstructure. The manufacturer has provided material values for the constituents (Table 1). Only one shear modulus is used in this paper and therefore ν_23f_ needs to be calculated using the given value of G_23f_ considering the isotropic condition in the 2-3 plane.

**Figure 4:**
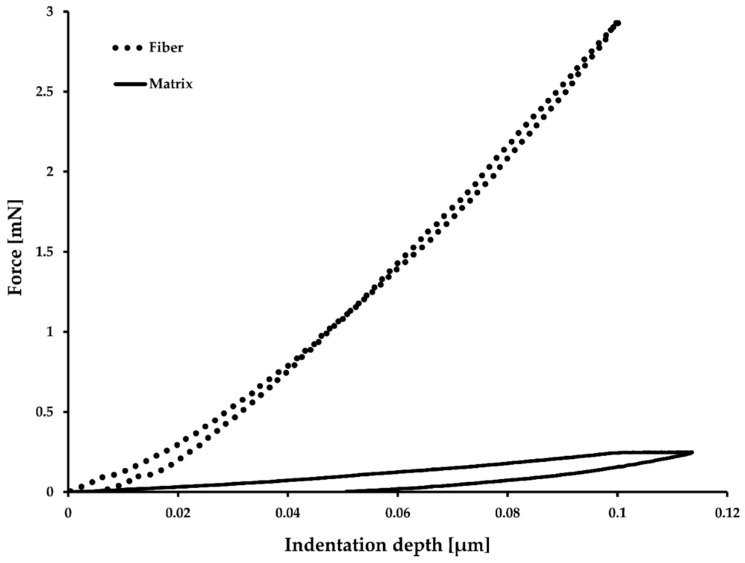
Loading/unloading curves of fiber and matrix from which indentation modulus is calculated.

Nanoindentation was conducted in order to verify some of the given parameters (Figure 4). There is a large discrepancy between the Young’s moduli obtained from indentation data and values reported by the composite manufacturer (Table 2). The reason for differing behavior in the case of the fiber is proposed to be nanobuckling and compressive failure in the nanostructure of carbon fiber [50]. Others have obtained similar results for polyacrylonitrile-based carbon fibers [37,51,52,53]. The resulting E_2f_ from these indentations is 13 GPa, which falls between the 20 GPa given by the manufacturer and inverse micromechanics [14,17] from transverse compression tests indicating E_2f_ should be 10 GPa. The reason for differing epoxy stiffness is attributed to the constraint imposed by surrounding fibers [53].

### 3.2. Microstructure

**Figure 5:**
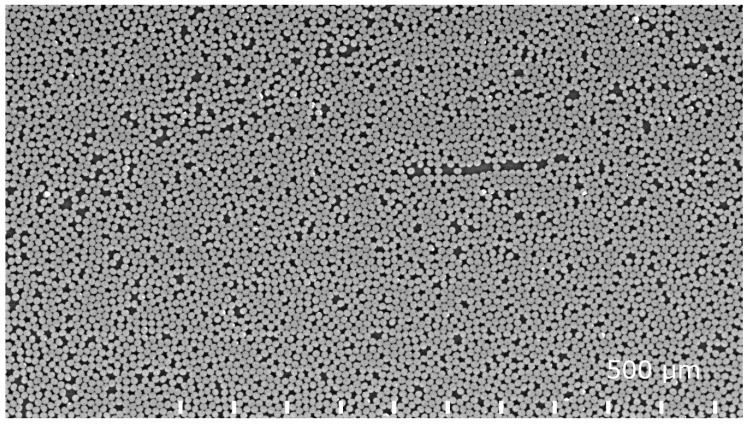
Electron backscatter image distinguishes between fiber and matrix due to a difference in average atomic number (Z contrast).

**Figure 6:**
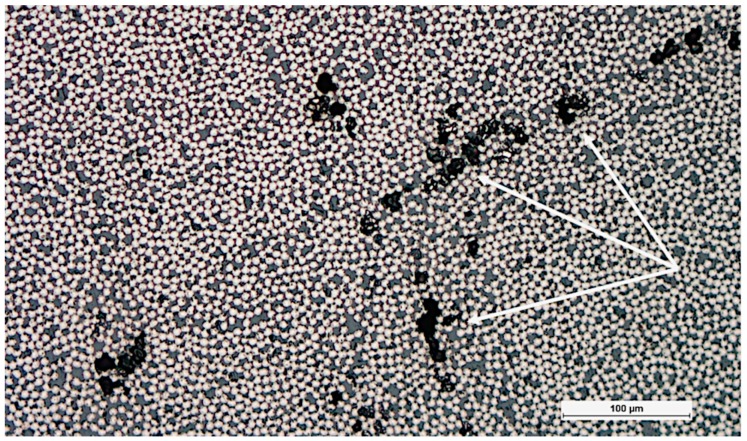
Optical micrograph showing pores (marked with white arrow).

The microstructure obtained using electron microscopy (Figure 5) was processed algorithmically into a multiphase mesh. The fiber volume fraction was also obtained from the segmenting process. The fiber volume fraction was found to be 0.65, which corresponds to the fill ratio disclosed by the manufacturer. The introduced pores were characterized using light optical microscopy (Figure 6). The image shows clusters of multiple pores approximately 20 µm in diameter.

### 3.3. Microscale Modelling

**Figure 7:**
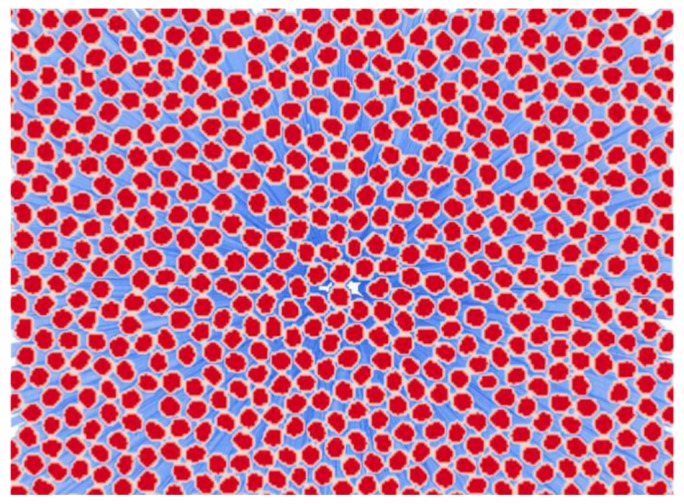
Image-based meshing of the microstructure showing a perspective view of the fiber and interphase.

The statistical representation of the microstructure meshed from the previous step (Figure 7) can be evaluated by looking at homogenised values as a function of volume fraction of total microstructure size. It is noted that stabilization of the prediction takes place at 0.5 of total volume, indicating that the system is representative with respect to property computation. In addition, it is noted that chosen boundary conditions for the simplistic description of material behavior do not markedly influence the computation, as no significant deviations in the predictions are visible as the volume fraction approaches 1.0.

A one-at-a-time sensitivity analysis was conducted using the micromechanical finite element model. In total, 14 simulations were made and the resulting homogenised composite properties were analyzed in terms of the five independent composite constants. The relative change to the reference value was calculated and all ratios between 0.9 to 1.1 were omitted as insignificant. Ratios below 0.7 are marked with red indicating a significant reduction while values above 1.3 are marked with green indicating a significant increase. Values in between are marked with yellow to indicate a small change (Table 3). The most important constituent properties are E_1f_, E_2f_, G_12f_ and E_m_ while the Poisson’s ratios do not have a strong effect on composite properties, especially when considering the realistic bounds for those values. E_1_ is affected by E_1f_ while E_2_ is affected by E_2f_ and E_m_ as shown also in the analytical formulation [20]. E_1f_ has a surprising effect on G_12_, which is not included in the analytical model, which, on the other hand, exaggerates the role of E_m_ on G_12_. The Poisson’s ratio ν_12_ is insensitive to all parameters while ν_23_ changes with many of the parameters.

### 3.4. Macroscale Modelling

**Figure 8:**
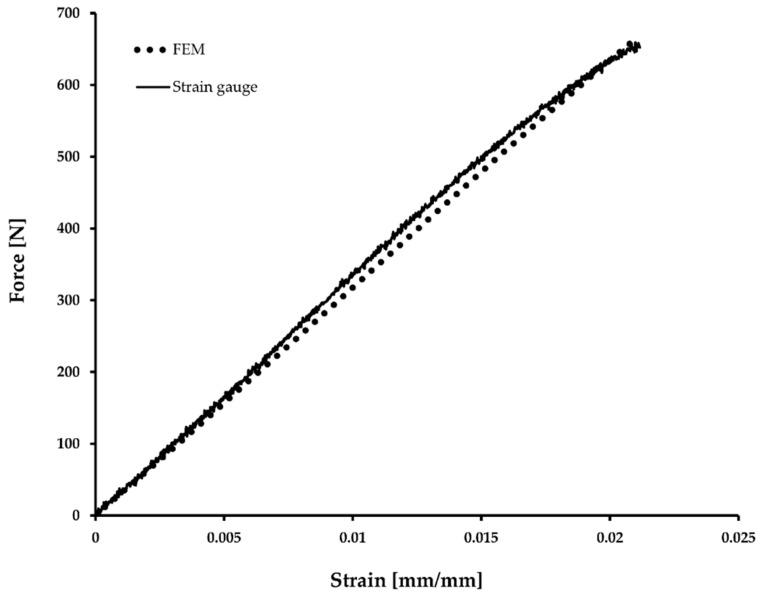
Three point bending response using finite element method and experimental mechanics.

Macroscale modelling was used to obtain relevant boundary conditions for the RVE, but also to confirm that the three-point bending simulation using homogenised material properties behaves correctly. The simulation results are in good agreement with measured strain gauge and force cell values (Figure 8), although the measurements show a non-linear dependency, which is not captured by the model. In a similar way to what was done for the microscale modelling, a one-at-a-time sensitivity analysis was conducted by doubling and halving the homogenised material constants. E_1_ could not be halved because the resulting displacements were too large for a stable solution to be found. Instead, a factor of 0.75 was chosen for the reduced E_1_ case. Looking at the relative maximum von Mises stress, strain in fiber direction and center roller displacement it appears like E_1_ is the main governing parameter in three-point bending, while E_2_ and G_12_ have only a weak effect on simulated component behaviour. E_1_ is affected only by E_1f_ (Table 3) and therefore E_1f_ is the only constituent property that needs to be known accurately for macroscale modelling the bending of UD CFRP beams. Even an isotropic assumption leads only to a 3% error, meaning that the anisotropy is not essential to be included in the model. However, these results are only for three-point bending and the transverse properties could play a larger role in other load cases.

The homogenised material properties were verified using various macroscopic experiments. Results obtained using uniaxial tensile testing, uniaxial compression testing, transverse compression, flexural testing, quasi-static elasticity imaging (QSEI), analytical formulas and homogenisation of the micromechanical finite element model are summarized in Table 4. G_12_ and ν_23_ are the only parameters where discrepancy is seen. The first was not directly measured and the latter was obtained from transverse compression where boundary conditions and specimen geometry, especially the length, could affect the results as well as the location of the virtual strain gauges on the cross-section.

### 3.5. Effect of Defects

**Figure 9:**
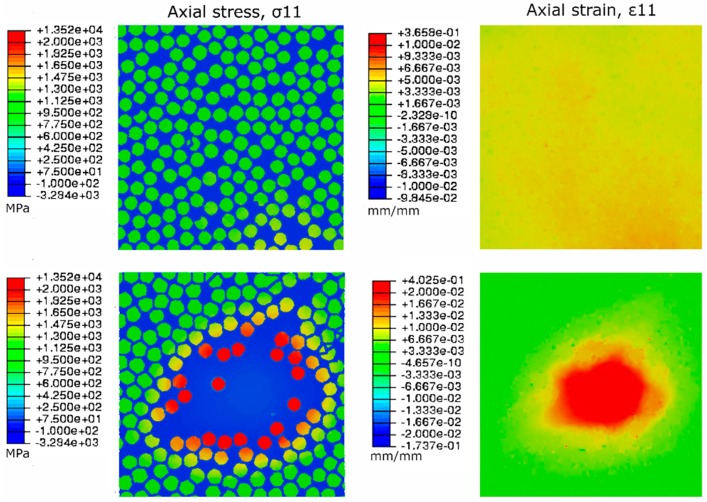
Comparison of reference microstructure (**top**) and resin-rich defect (**bottom**) in tension-dominated loading.

**Figure 10:**
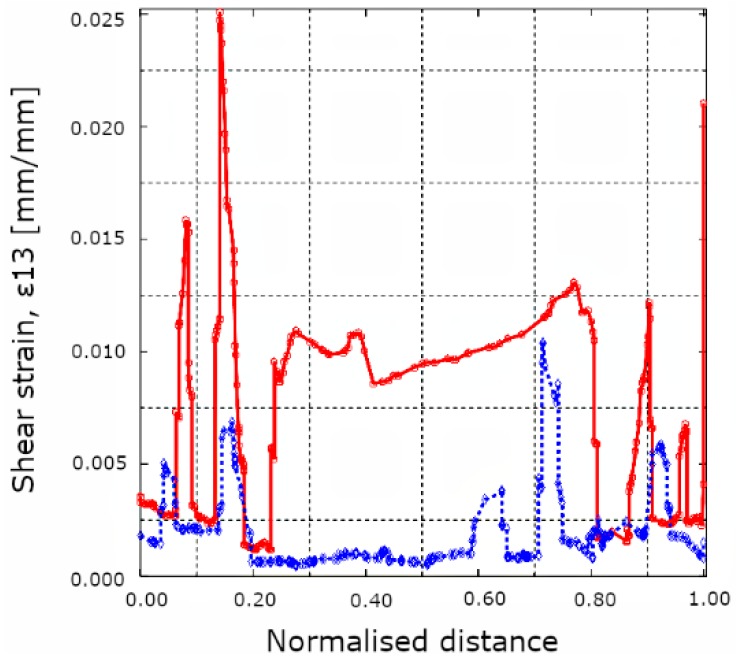
Line plots of the shear strains at the center (red) of the resin-rich microstructure and at the bottom (blue) in a shear-dominated load case.

The last step of the multiscale modelling approach is to use the boundary conditions obtained from macroscale hot spot analysis for loading an RVE with a known defect. A resin-rich area in the microstructure is used to represent the microstructure with porosity (Figure 6). The lack of reinforcement fibers causes a local increase in strain and the surrounding fibers have to carry the load (Figure 9). However, this type of matrix defect does not adversely affect the measured flexural strength (Table 5). On the other hand, apparent ILSS tests indicate that porosity reduces the shear strength of the CFRP material and failure occurs in the center plane (1-3) of the specimen. Changing the RVE load case to correspond with the failure location observed in ILSS tests and looking at the shear strain components shows the effect of the resin-rich zone (Figure 10). As expected, heterogeneities in the microstructure cause local effects that can be quantified using the modelling approach presented here.

## 4. Conclusions

A micromechanics-based model was created to estimate the effect of defects in unidirectional carbon fiber composites. The modelling approach requires the following inputs: fiber and resin properties, microstructure, macroscale load cases, boundary conditions and defect morphology and location.

- Fiber and resin properties given by manufacturers should be taken with caution, considering the measurement methodology is not known. Determining constituent properties with nanoindentation gives poor results for both longitudinal fiber and matrix properties. The phenomena could be attributed to nanoscale buckling of the fiber and a constraint effect in the matrix, respectively. Transverse nanoindentation results of the fiber were closer to inverse micromechanics solutions and literature values of similar fibers. Despite the uncertainty in input parameters, the homogenised composite properties were in good agreement with experimental verifications.
- SEM images were successfully segmented algorithmically enabling the generation of a representative mesh of the microstructure. However, the use of 2D micrographs omits fiber waviness effects and defect morphology.
- Macroscale simulations were in good agreement with experimental work both in terms of elastic response and failure location. A sensitivity analysis showed that only the longitudinal modulus of the composite plays a significant role in the macroscale response. Furthermore, that parameter is mainly affected by the longitudinal fiber modulus, which is typically known. In fact, transverse properties are insignificant to a degree where an assumption of isotropic material properties leads only to a 3% error in stress/strain. However, three-point bending was the only load case used.
- A batch of high-porosity material was produced and the effect of porosity was simulated using a resin-rich area in the microstructure. The simulations show stress and strain concentrations in the fibers and matrix due to the heterogeneous microstructure. However, long-beam bending experiments showed no difference in strength between reference and porous samples. This can be attributed to the load sharing mechanism of unidirectional reinforcement fibers in predominantly tensile loading. However, short-beam bending experiments showed a 20% reduction in apparent shear strength for the samples with porosity. The effect of matrix defects on shear strength was simulated by looking at the shear strains in the center plane where failure occurred. The results depict a highly strained matrix at the resin-rich zone compared to a homogeneous microstructure.

The approach presented here can be used to estimate the residual strength of a component with a known microscale defect. The component and load case used in this paper is simple and future work should include validating the approach for other component geometries and loads.

## Figures and Tables

**Table 1:** Material constants given by the manufacturer.

Constituent	E_1_ (GPa)	E_2_ (GPa)	G_12_ (GPa)	G_23_ (GPa)	ν_12_	ν_23_ (Calculated)
Fiber, f	239	20	30	8	0.2	0.25
Matrix, m	3.2	-	-	-	0.35	-

**Table 2:** Comparison of indentation results with the given reference values.

Location	Contact Stiffness, S (mN/µm)	Indentation Modulus, M (GPa)	Young’s Modulus, E (GPa)	Reference, E (GPa)
Fiber, long.	46 ± 4	50	55	239
Fiber, trans.	17.5 ± 0.9	19	13	20
Matrix	13 ± 3	11	13	3

**Table 3:** Sensitivity of homogenised composite properties to fiber and resin properties.

Property	Multiplier	E_1_	E_2_	G_12_	ν_12_	ν_23_
E_1f_	0.5	↓ 0.5	-	→ 0.8	-	-
E_1f_	2	↑ 2	-	→ 1.2	-	-
E_2f_	0.5	-	↓ 0.6	-	-	↑ 1.4
E_2f_	2	-	↑ 2.2	-	-	→ 1.2
G_12f_	0.5	-	-	→ 0.7	-	-
G_12f_	2	-	-	↑ 1.7	-	-
ν_12f_	0.5	-	-	-	→ 0.8	↑ 1.4
ν_12f_	2	-	-	-	→ 1.2	↓ 0.1
ν_23f_	0.5	-	-	-	-	→ 0.8
ν_23f_	2	-	-	-	-	-
E_m_	0.5	-	↓ 0.5	-	-	↓ 0.1
E_m_	2	-	↑ 1.9	→ 1.2	-	→ 1.3
ν_m_	0.5	-	-	-	→ 0.8	-
ν_m_	2	-	-	→ 0.8	-	→ 1.3

↑ Large increase; → Small change; ↓ Large decrease.

**Table 4:** Summary of simulated and measured composite properties.

Method	E_1_ (GPa)	E_2_ (GPa)	G_12_ (GPa)	ν_12_	ν_23_
Uniaxial tension	148	-	-	0.29	-
Uniaxial compression	135	-	-	0.29	-
Transverse compression	-	7	-	-	0.5
Flexural testing	152	-	-	-	-
QSEI [46]	155	7	10	0.29	-
Analytical [20]	156	10	5.3	0.25	0.29
RVE FEM	158	9	6.8	0.25	0.34

**Table 5:** Comparison of strengths in defective and pristine samples.

Material	Flexural Strength (MPa)	Apparent ILSS (MPa)
Reference	3120 ± 30	93.9 ± 0.2
Porous	3150 ± 132	73.1 ± 3.1
